# The Personality of the Physician

**Published:** 1908-04-25

**Authors:** Isabella Mears


### The General Practitioner's Column.

[Contributions to this Column are invited, and if accepted, will be paid for.]

### THE PERSONALITY OF THE PHYSICIAN.

By ISABELLA MEARS, L.R.C.P.I.

An address delivered before the Debating Society of the Medical College for Women, Edinburgh.

The present-day system of medical education leads us more and more to believe and to trust in the material. We are learning day by day the extreme importance of the things that can be ascertained only by eye, by ear, and by touch. The microscope, the stethoscope, the test-tube, and the dissecting knife reveal to us many wonders. We may perhaps be pardoned if we forget for a time that all these wonderful facts of anatomy, physiology, and chemistry, all these manifold theories of diseases, and all the newest methods for the radical treatment of disease, are not, and cannot ever be, the whole round sum of the equipment of a medical man or a medical woman. But, above and beyond all the knowledge and skill that can be imparted by the colleges and the universities and acquired by the student, the physician must possess qualities of character that make for healing, without the possession of which no amount of learning, of skill with the knife, or of quickness of observation and of diagnosis, can make him or her truly successful and of real value in helping a patient to regain his health.

If you look at the daily life and practice of the leading surgeons and physicians—it would be invidious to mention names, though to point my moral I should like to do so—you will observe that the man whose fame goes forth through his old patients and his old pupils to the very ends of the earth is the man whose skill is evident, whose aptitude with the knife it is a delight to watch, who has an eagle eye for the detection of obscure disease, whose simple questions seem to turn the patient with his ailments inside out before your wondering eyes. But he is more than a skilful investigator and clinician; he is a man of bright and alert nature; a man of quick sympathy and of kindly deed; a man who is thinking more of the patient and of the student than of his own ease, or even of his own fame; and who is giving daily, out of the storehouse of his personality, fresh supplies of strength to cope with difficulties, fresh supplies of patience for research into the deep things of nature, and, not least, fresh supplies of courage and of hope by which the patient is led to exert himself to help on the curative processes that are being established in his system.

This quality, the personal influence of the physician over the patient, is the subject of my talk with you to-day; it is the influence of one personality over the minds and personalities of others, causing them to change towards good or evil, towards sinfulness of nature and disease of body, or towards health and strength of mind and body alike.

You can see the effects of the same law, call it what you will, "the unconscious influence of personality," or "the instinct of imitation," in all the conventionalities of life. The arbitrary fashions of dress, the mode and arrangement of houses, of hours, and of meals; the enthusiasm of a crowd, the unreasoning panic of a crowd in face of a possible danger; the contagion of a certain class of crime, as when there is a run of suicides, homicides, burglary, garrotting, or poisoning; or the ecstasy of great crowds during a revival of religion—all these things show that the unconscious influence of personality is a main factor in moulding civilisation, in making a whole community conform to a common standard of thought or action.

It is not easy for one individual to stand out alone against popular feelings and ideas, but at various times in the history of the nations there have been men and women who have been able, by force of mind, by originality of character, or by sheer strength of will, to sway the multitudes, and through the influence of such individuals great reforms have been brought to pass. It is not, perhaps, for us to hope for such achievement in our life, but for all that we have a right to consider the matter, and to remember that each one of us has this great gift of personality in our own possession, to be used for good or for ill.

The power of personal influence has been often used in the cure of disease. There have been numberless quacks who, without the help of any medical training, have used this power with varying success. I fancy that we generally hear of their successes, but not of their failures. Then we read of women, like Mrs. Eddy and Mrs. Helen Wilmans, who have practised the use of the power of personal influence, and who, by no other apparent means, have been able to change the outlook and raise the thoughts of sick people in such a manner that they have been able to lay aside their illnesses, and have become to all appearance healthy, normal individuals. It seems a pity that men and women who have accurate scientific knowledge of the human frame and of disease should entirely scoff at and ignore the power of such a factor in the treatment of their patients. The fact is that all doctors do employ it more or less unconsciously, and that a few employ it consciously and successfully. You will hear many a patient say: "It does me good just to see my doctor; I am always better after his visit." Again, you will find a patient weak and depressed because the doctor expressed an unfavourable opinion during his morning visit. To my own knowledge, patients have died within a few days of hearing the verdict "There is no hope for you"; while other patients will date the turning-point of an illness from the day when the consultant came with a bright face and a cheerful prognosis.

The patient of weak nervous system is always with us. He lacks the power of inhibition of the emotions and of the judgment. He is impressionable, and depends for guidance upon the will of others. He does not know how to use his own strength of will for the purpose of recovering his health, and he will sometimes even show a deliberate wish to evade the means used for cure. Along with weakness of body, from whatever cause arising, there is frequently a marked despondency of mind, which militates against the progress of a cure. There is also, almost constantly, a loss of staying power, the patient being unable to stand anxiety or mental strain, or even to anticipate a change of circumstance, without suffering for it. But the symptom that is more difficult than all others to deal with is that of self-engrossment, when the attention of the patient is directed wholly inwards. He can then talk or think of nothing but himself; all the world centres round the "ego"; all topics of conversation lead back to the eternal round of bad nights, of fears, of pains and aches. I have known such a patient talk straight on for an hour by the clock, retailing symptoms imaginary or real, going round and round the subject of self, of past miseries and present woes, with an occasional prognosis of future evils, and then he was only stopped by the plea of "No time for more to-day"; but he could have gone on.

Such symptoms are not those of lunatics, of patients who would be beyond the sphere of most of you. They are ordinary symptoms that occur when the nervous system of a patient is below the normal. All of them come under the observation of any family doctor who is doing ordinary practice. They do not readily yield to ordinary medicinal treatment, but they can be controlled and ameliorated by the personal influence of the doctor and the nurse, and they disappear as the patient's nervous tone is gradually restored to its normal condition.

I have used these illustrations for the purpose of showing you how important it is for each medical woman consciously to cultivate her power of personal influence. The question now is, "How may a student of medicine do this? How shall she learn to cultivate and improve her personality? And how shall she learn to bring this power to bear upon others for their good?" The subject of the cultivation of the personality is a very wide one. In the few minutes that remain I cannot do more than bring some wide generalisations before you. Personality, by which I mean "a character that can be used in personal influence for the good of others," is not a thing that is a gift or a miracle; it does not come to us spontaneously; it has to be built up by endeavour, by thought, by daily choosing, by daily doing. Each individual has more or less control over his development. He can choose his environment, his work, and his books. He can so train his eye and his intelligence as to see and observe beauty in many things that to other people are quite commonplace. More than that, the individual can to a great extent choose what he shall hear; he can choose his friendships, and we all know what an influence our friendships have upon our character, how the words of a friend remain with us and print their marks upon our memory. Thus are the gates of our citadel always open to receive supplies, and daily, nay, momently, impressions, facts, and thoughts are crowding in to help us to build up and beautify the dwelling-place of our individuality. Every sight we see, every word we hear, every person we come in contact with, leaves an impress upon our own character. It is for us, then, to choose our environment, to choose our books, and to choose friends that will help us to make our personality useful as well as beautiful.

But we must remember that there is also work to do inside our citadel, and here again comes into power the same law of the action of presence, for by the knowledge that we use, by the thoughts that we cherish, and by the emotions that we allow to dwell within us, are our characters built, strengthened, or weakened.

There are philosophers who tell us that thoughts are the only real things. Thinking comes before creating. "God thought about me, and so I grew," George Macdonald tells us. Certainly, thinking has great power to build up the palace of the soul, and the woman who has learned to think, to set apart a bit of time from every busy day for quiet thought, and who has persevered in doing this, has found a source of enduring happiness, a cure for worry, a well of strength. She has learned to open the doors to the infinite, whence come stores of love, of strength, of peace, and of wisdom.

Thoughts and emotions lead to action, and here comes the point of the whole matter, which is the action of our presence, our personal influence upon others. For it is only by being strong that we can communicate strength to others; it is only when we are filled with love that we can give out compassion and sympathy to the suffering; it is only when we know how to "get wisdom" that we can guide and comfort the erring, and bring them back again into the right way.

The sum of the whole matter is that, when the thoughts that indwell us are good thoughts; when the heredities, the environments, the circumstances of our lives do not control us, but we rise above them; when we build up, by the action and reaction of our feelings, of our thoughts and of our deeds, a beautiful character, steadfast and constant, calm and resourceful; then, and then only, shall we fulfil our destiny as helpers and healers of those who suffer from weakness and from disease.

